# General side effects and challenges associated with anti-epilepsy medication: A review of related literature

**DOI:** 10.4102/phcfm.v12i1.2162

**Published:** 2020-06-30

**Authors:** Ngonidzashe Mutanana, Maria Tsvere, Manase K. Chiweshe

**Affiliations:** 1 Department of Child Sensitive Social Policies, Faculty of Social and Gender Transformative Sciences, Women’s University in Africa, Harare, Zimbabwe; 2 Department of Development Studies, Institute of Lifelong Learning and Development Studies, Chinhoyi University of Technology, Chinhoyi, Zimbabwe; 3 Department of Sociology, Faculty of Social Sciences, University of Zimbabwe, Harare, Zimbabwe

**Keywords:** anti-epilepsy medication, general side effects, psychological challenges, social challenges, economic challenges

## Abstract

**Background:**

This study is coming against the background of people with epilepsy who are abandoning anti-epilepsy medication in developing countries, such as Zimbabwe.

**Aim:**

The aim of this article was therefore to review the general side effects and challenges associated with these anti-epilepsy medications.

**Setting:**

The researchers reviewed literature related to the general side effects, psychological, social and economic challenges associated with anti- epilepsy medication.

**Methods:**

To answer the research questions, the researchers used a narrative approach.

**Results:**

Findings of the study reflected that the general side effects associated with anti- epilepsy medication include feelings of tiredness, stomach upset, dizziness or blurred vision, which usually happen during the first weeks of taking medicines. Psychologically, an individual with epilepsy may suffer cognitive problems that are associated with thinking, remembering, paying attention or concentrating and finding the right words to use. Socially, people with epilepsy experience social isolation, dependent behaviour, low rates of marriages, unemployment and reduced quality of life. Using anti-epilepsy medication is also associated with economic challenges.

**Conclusion:**

The researchers concluded that some people with epilepsy have discontinued using anti-epilepsy medications because of these side effects and challenges.

## Introduction

Education and counselling is an important subject matter in epilepsy management. It could be the reason why there is a big treatment gap in developing countries. As observed by Mutanana,^1^ patients are not well informed about challenges associated with epilepsy medication, which explains why they opt for traditional medications especially if they suffer from the general side effects and challenges associated with anti-epilepsy medications of the developed world. This study therefore sought to analyse the general side effects and challenges associated with anti-epilepsy medication.

The work of the 19th-century English neurologist John Jackson marks the beginning of modern medical era of epilepsy.^2^ The author adopts Jackson’s definition of a seizure: an occasional, excessive and disorderly discharge of the nerve tissue or muscles. For Fisher et al.,^3^ an epileptic seizure is a transient occurrence of signs or symptoms because of abnormal excessive and/or synchronous neuronal activity in the brain. Both definitions imply the same thing, excessive or disorderly muscles among people with epilepsy that is linked to the brain activity. Cherney^4^ also identified non-motor seizures. According to the author, these are normally called absence seizures and are typical of absence seizures. An individual with non-motor seizures may have brief twitches that can affect a specific part of the body or just the eyelids. Cherney^4^ posted that epilepsy causes the brain to send abnormal signals and this activity results in seizures. Fisher et al.^3^ conclude that the definition of epilepsy requires the occurrence of at least one epileptic seizure. These seizures happen because of a number of reasons such as injury or sickness. Cherney^4^ thus describes epilepsy as a condition that causes recurrent seizures and is treated with anti-epilepsy medication.

Tuan^2^ argues that effective anti-epilepsy medications have been available since 1850 when bromide was first introduced. These anti-epilepsy medications are selected according to the patient’s type of seizures and other individual characteristics. There are more than 20 types of anti-epilepsy medications available and one’s option depends with age, lifestyle, and type of seizure and how often he or she has seizures.^5^

Cherney^4^ believes that the most common way to treat epilepsy is with anti-epilepsy medication. Findings have revealed that they allow up to 60% – 70% staying free from seizures and are less harmful because they are scientifically proven, unlike traditional modes of anti-epilepsy treatment.^4^ Similarly, Glauser et al.^6^ and Glauser et al.^7^ observed that anti-epilepsy medications provide the best quality of life with no seizures and fewest adverse effects from treatment. What it shows is that anti-epilepsy medications are useful in treating epilepsy, even though they have been condemned in Africa for various reasons to be discussed below.

Jilek-Aall and Rwiza^8^ also confirm a follow-up of anti-epilepsy medication study in Tanzania, which revealed that about 52.4% of epileptic patients managed to achieve complete seizure with the drugs, with 36% reducing epilepsy frequency of seizures, while only 7.9% experienced no change during their 20 years of treatment. Nimaga et al.^9^ report that in rural Mali, about 80% of the 96 patients treated with phenobarbital became seizure free within 1 year. The general belief among medical practitioners is that epilepsy can be treated or controlled. What it shows is that treatment of epilepsy is easy because most people with epilepsy can be managed without sophisticated investigations. While it has been noted that epileptic seizures could be controlled with medications such as phenobarbital, carbamazepine and phenytoin in 70% of the patients, Dewa^10^ observed that the effectiveness of all medicines including anti-epilepsy medication depends on adherence to the whole treatment process. This includes taking of medicines’ required quantities and timely going for medical or health reviews as appointed. However, Epilepsy Support Foundation Zimbabwe^11^ has indicated that about 86% of people living with epilepsy are not on anti-epilepsy medication in Zimbabwe, implying that they are not taking these anti-epilepsy medications. What then are the side effects and challenges associated with these anti-epilepsy drugs?

## Research objectives

The aim of this study was to analyse the general side effects and challenges associated with anti-epilepsy medication. The following were the specific objectives of the study:

1. to identify the general side effects associated with anti-epilepsy medication
2. to examine the psychological, social and economic challenges associated with anti-epilepsy medication.

## Research questions

1. What are the general side effects associated with anti-epilepsy medication?
2. What are the psychological, social and economic challenges associated with anti-epilepsy medication?

## Research method

To answer the above research objectives, the authors used a narrative approach in reviewing the literature. A narrative review basically summarises a body of literature and draws conclusions about the topic.^12^ When conducting a narrative literature, the author must have a sufficiently focused research question.^13^ The question that the authors sought to answer in this study was: What are the side effects and challenges associated with anti-epilepsy medications? This model followed a systematic data processing approach that comprised the following three steps: (1) literature searching and screening, (2) data extraction and data analysis and (3) writing of the literature review.

## General side effects associated with anti-epilepsy drugs

Many people are concerned with side effects associated with epilepsy medication, particularly because they affect the quality of their life. Anti-epilepsy medications may cause unwanted side effects in some patients. The common side effects identified by Stephen et al.^14^ are feelings of tiredness, stomach upset, dizziness or blurred visions, which usually happen in the first few weeks of taking seizure medicines. According to Epilepsy Scotland,^15^ these drugs may also cause fatigue, nausea, urinary retention and sexual dysfunction. If patients with epilepsy are not educated on these side effects, they eventually abandon epilepsy medication and attempt traditional medication.

Stephen et al.^14^ also explain some general side effects associated with anti-epilepsy medications such as allergic reactions, for instance, rash. Karceski^16^ also identified increased sexual dysfunction, diminished fertility and disruption of the normal menstrual cycle as some of the side effects associated with anti-epilepsy medication. Mutanana^1^ observed cases of women with epilepsy who are failing to conceive, while several men are experiencing erectile problems after taking anti-epilepsy medication. For children, the general side effects are drowsiness in class, inattention and restlessness, which affect normal brain functioning and can make learning to be even more difficult.^17^

The anti-epilepsy medications will cause teratogenicity leading to miscarriages or children with disability. This also applies to men who become sexually dysfunctional because of medication. The majority of them are young, and sexual dysfunctionality has an effect on their marriages. In his study on challenges associated with epilepsy medication, Mutanana^1^ observes that people with epilepsy are not well informed about these side effects when they begin to take anti-epilepsy medications. Consequently, some will opt for traditional medications as a plateau to these side effects. As such, if the doctor recommends anti-epilepsy medication, he or she should discuss the benefits and side effects of the medication.^1^ This will help people with epilepsy to appreciate the challenges associated with the medication.

## Psychological challenges associated with anti-epilepsy medication

Anti-epilepsy medication can affect how the brain works in some cases. These anti-epilepsy medications lower excitability of nerve cells in the brain and this affects normal activity.^14^ Cognitive problems are problems associated with thinking, remembering, paying attention or concentrating, and finding the right words, and can be because of side effects of anti-epilepsy medications. A study by Al-Faris et al.^18^ in Saudi Arabia discovered that forgetfulness contributed to about 22.5% of the reasons why children fail to attend epilepsy treatment review visits. In other words, these anti-epilepsy medications may affect the cognitive function of the person with epilepsy. As such, some people with epilepsy may abandon anti-epilepsy medications because of these challenges.

Joseph et al.^19^ posited that epilepsy and its treatment may affect the way that people with epilepsy think and behave. This affects the memory, language, planning and reasoning among people with epilepsy. This may affect how a person with epilepsy relates with family members and workmates. Some anti-epilepsy medications also affect a person’s energy level, mood, motivation or how fast they think or perform a task.^14^ Rodriquez^20^ also observed that anti-seizure medications like phenobarbital can affect the patient’s mood and increase depression. Mitchel^21^ posted that cognitive, psychiatric and behavioural abnormalities in children with epilepsy are attributable to antiepileptic medications. Rodriquez^20^ discovered that anxiety can also occur as a reaction to the diagnosis and some side effects of anti-epilepsy medication. Feeling socially isolated because of epilepsy also influences anxiety symptoms. Mitchel^21^ claims there is an increasing concern about suicidal ideation in older children and youth treated with anticonvulsants. Patients with severe mood disorders are also prone to develop suicidal ideation.

Epilepsy medication restricts activities, for instance, taking medicines, not driving, maintaining regular sleep cycles, limiting alcohol changes and the lifestyle, and can lead to a loss of independence.^20,22^ Mitchel^21^ observed that most sedative drugs have the potential for causing excitement and agitation. She gives an example of the phenobarbital anticonvulsant drug that she claims may cause sustained behavioural difficulties such as over reactivity, irritability and disturbed sleep. In her analysis, 5% – 25% of children experience over-reactivity because of phenobarbital. Bazil^23^ also supports Mitchel when he claims these anti-epilepsy drugs may result in insufficient sleep on top of inadequate sleep hygiene, coexisting sleep disorders and circadian rhythm disturbances.

## Social challenges associated with anti-epilepsy medication

Use of anti-epilepsy medication is also associated with social isolation, dependent behaviour, low rates of marriage, unemployment and reduced quality of life.^24,25,26^ Mitchel^21^ observed adverse outcomes that include less frequent marriages, employment, skilled occupations and social isolation. In his study in Finland, Mitchel^21^ claims 60% participants were independent in activities of daily living. In Sweden, she states young adults with persisting absence seizures were more likely to be employed in an unskilled job or in an occupation below expectations for educational level as compared to peers without epilepsy. Mitchel^21^ also discovered that social isolation was reported in 34.5% as compared to 7.9% of the reference group and 74% reported that epilepsy had affected at least one area of their social functioning.

Some studies have examined the gender issue in Asia. In Pakistan, Aziz et al.^27^ observe that female patients with epilepsy were failing to cope with the pressure from society and family, and consequently tend to internalise the prejudice and discrimination. In Sri Lanka, Gamage^28^ discovered that men are not willing to accept women with epilepsy and that epilepsy is a valid reason for divorce. They believe that women with epilepsy cannot bear children. In Korea, statistics show that about 24.5% have been discriminated at their workplace.^29^ More than half of those who have disclosed their illness have been refused a job because of epilepsy.

Epilepsy Support Foundation^30^ also argues people with epilepsy in Zimbabwe face a number of challenges. These include lack of understanding of the disease and impaired access to treatment as well, predisposition to burns and injuries, and prejudice that also affect socialisation, marriage, school and work resulting in impaired quality of life and socioeconomic status. Mugumbate and Nyanguru^31^ also identify overprotection as another challenge that people with epilepsy face. They suffer from overprotection and have some difficulties in securing an independent life.

## Economic challenges associated with anti-epilepsy medication

Use of anti-epilepsy medication is associated with economic burden.^24,25,26^ The development of new anti-epilepsy medication is costly and risky. Perucca et al.^32^ and Lalonde et al.^33^ posted that chances for successful completion of development and the approval by the regulatory authorities are less than 10%, even for those drugs which are in phase 1 stage. Despite the funding of antiepileptic development, Wahab^34^ states that these anti-epilepsy medications lack safety and efficacy, and historically many of these drugs have been withdrawn from the market because of their severe adverse effects. Newly developed anti-epilepsy medications are expensive and their lifelong use makes it almost impossible to afford for the people in developing countries such as Zimbabwe.^34^

Studies have also revealed that these anti-epilepsy medications are normally in short supply^35^ because of an under-resourced healthcare system. Chilopola et al.^36^ have also identified distance (from community to hospital) and lack of knowledge as some challenges associated with anti-epilepsy medications. Mugumbate^37^ also identifies economic aspects that affect people with epilepsy. In his findings, 93% indicated securing adequate income as a challenge, and consequently they fail to acquire anti-epilepsy medication. The majority of those who are diagnosed to be epileptic cannot afford to buy these anti-epilepsy medications and this has an effect on compliance.

## Discontinuation of anti-epilepsy medication

**FIGURE 1:**
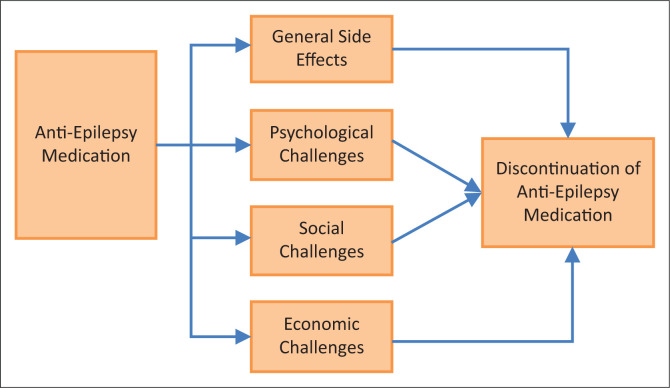
Factors associated with discontinuation of anti-epilepsy medication.

Studies in some tribes of Central and South America, in the southern-east part of Asia and Africa, indicate that they continue to perpetuate traditional beliefs about epilepsy.^38^ Epilepsy is frequently thought as a punishment for evil deeds. In some cases, it is viewed as causation for breaking certain taboos. These traditional beliefs prevent people with epilepsy from seeking anti-epilepsy medication. Khadilkar^39^ further asserts that some people with epilepsy discontinue treatment because of fear of the general side effects associated with the medication. In India, for instance, Das et al.^40^ reported 43% discontinuation rate within 1 year. Kadilkar^41^ blames discontinuation of anti-epilepsy medication on high cost of treatment, superstitions and cultural beliefs, but Bharucha^42^ believes dimensions of medical, social, psychological and financial consequences of epilepsy are enormous and a cause for discontinuation of anti-epilepsy medication. A large proportion of people with epilepsy in the developing community, as reported earlier, do not get anti-epilepsy medication. Figure 1 is a diagrammatic presentation of anti-epilepsy medication and challenges associated with it.

Mitchel^21^ points out that people with epilepsy, parents and teachers are well read over the adverse effects of medications. There are frightening stories in lay media and on the Internet about epilepsy medication. Mitchel^21^ believes some people may be under the wrong impression regarding these drugs and expresses the fear that a medicine will make them retarded or cause developmental problems. Eventually, these fears may lead people with epilepsy to avoid administering prescribed medications. Mutanana^1^ also observes that some people with epilepsy do not use anti-epilepsy medications because of the challenges associated with these medications.

### Ethical consideration

This article followed all ethical standards for a research without direct contact with human or animal subjects.

## Conclusion

From these findings, the authors concluded that there are general side effects associated with anti-epilepsy medication. These include feelings of tiredness, stomach upset, dizziness or blurred visions, which usually happen in the first few weeks of taking seizure medicines. Psychologically, there are cognitive problems associated with thinking, remembering, paying attention or concentrating, and finding the right words, which can be because of side effects of seizure medicines. Socially, use of anti-epilepsy medication is also associated with social isolation, dependent behaviour, low rates of marriage, unemployment and reduced quality of life. Economically, use of anti-epilepsy medication is associated with economic burden. These general side effects and challenges are associated with discontinuation of anti-epilepsy medication.

## Recommendations

Based on these conclusions, the authors recommend healthcare providers to ensure clear, accurate and timely information to people with epilepsy and their families. Patients need to be educated about the general side effects associated with anti-epilepsy medication. Education and counselling needs vary across lifespan. For children and adolescents, they need to be educated on how to manage seizures at school and common learning problems, dealing with fears, school and vocational planning, establishing health habits, drugs and alcohol, transition to adulthood and impact of epilepsy on family dynamics. As for adults, they need education and counselling on career and vocational concerns, discussions with employers, driving regulations and transportation concerns, sexual and gender specific topics, drug–alcohol interactions, independent living and impact of epilepsy on relationships and family dynamics. Seniors too need to be educated and counselled on medication side effects, adverse interactions, adherence, drug–alcohol interactions, independent living and safety.

Psychologists and counsellors are also recommended to help people with epilepsy with their cognitive problems that are associated with thinking, remembering, paying attention and poor concentration. Epilepsy advocates should also go around educating communities about epilepsy to avoid social challenges. The governments, through their social departments, and non-governmental organisations should also assist with medication for people with epilepsy.

From the foregoing, it can be noted that education and counselling is an important subject matter in epilepsy management. It could also be the reason why there is a big treatment gap in developing countries. As observed by Mutanana,^1^ patients are not well informed about challenges associated with epilepsy medication, which explains why they opt for indigenous methods especially if they suffer from the general side effects of the medication. Tuan^2^ suggests establishment of community-based mental health centres to offer psychosocial support for people with epilepsy. Mental health centres should have the responsibility to provide care for people with epilepsy in the community.
